# PJA1 Coordinates with the SMC5/6 Complex To Restrict DNA Viruses and Episomal Genes in an Interferon-Independent Manner

**DOI:** 10.1128/JVI.00825-18

**Published:** 2018-10-29

**Authors:** Wei Xu, Chunqiang Ma, Qi Zhang, Rong Zhao, Dan Hu, Xuewu Zhang, Junbo Chen, Fang Liu, Kailang Wu, Yingle Liu, Jianguo Wu

**Affiliations:** a State Key Laboratory of Virology, College of Life Sciences, Wuhan University, Wuhan, China; b Institute of Medical Microbiology, Jinan University, Guangzhou, China; University of Southern California

**Keywords:** RING finger protein PJA1, SMC5/6 complex, DNA topoisomerases, hepatitis B virus, HBV, herpes simplex virus 1, HSV-1, episomal DNA, extrachromosomal DNA, host immune response

## Abstract

Viral and episomal DNAs, as signs of infections and dangers, induce a series of immune responses in the host, and cells must sense foreign DNAs to eliminate the invaders. The cell nucleus is not “immune privileged” and exerts intrinsic mechanisms to control nuclear-replicating DNA viruses. Thus, it is important to understand the action of viral DNA sensing in the cell nucleus. Here, we reveal a mechanism of restriction of DNA viruses and episomal plasmids mediated by PJA1, a RING-H2 E3 ubiquitin ligase. PJA1 restricts the DNA viruses hepatitis B virus (HBV) and herpes simplex virus 1 (HSV-1) but not the RNA viruses enterovirus 71 (EV71) and vesicular stomatitis virus (VSV). Similarly, PJA1 inhibits episomal plasmids but not chromosome-integrated reporters or endogenous genes. In addition, PJA1 has no effect on endogenous type I and II interferons (IFNs) and interferon-stimulated genes (ISGs), suggesting that PJA1 silences DNA viruses independent of the IFN pathways. Interestingly, PJA1 interacts with the SMC5/6 complex (a complex essential for chromosome maintenance and HBV restriction) to facilitate the binding of the complex to viral and episomal DNAs in the cell nucleus. Moreover, treatment with inhibitors of DNA topoisomerases (Tops) and knockdown of Tops release PJA1-mediated silencing of viral and extrachromosomal DNAs. Taken together, results of this work demonstrate that PJA1 interacts with SMC5/6 and facilitates the complex to bind and eliminate viral and episomal DNAs through DNA Tops and thus reveal a distinct mechanism underlying restriction of DNA viruses and foreign genes in the cell nucleus.

**IMPORTANCE** DNA viruses, including hepatitis B virus and herpes simplex virus, induce a series of immune responses in the host and lead to human public health concerns worldwide. In addition to cytokines in the cytoplasm, restriction of viral DNA in the nucleus is an important approach of host immunity. However, the mechanism of foreign DNA recognition and restriction in the cell nucleus is largely unknown. This work demonstrates that an important cellular factor (PJA1) suppresses DNA viruses and transfected plasmids independent of type I and II interferon (IFN) pathways. Instead, PJA1 interacts with the chromosome maintenance complex (SMC5/6), facilitates the complex to recognize and bind viral and episomal DNAs, and recruits DNA topoisomerases to restrict the foreign molecules. These results reveal a distinct mechanism underlying the silencing of viral and episomal invaders in the cell nuclei and suggest that PJA1 acts as a potential agent to prevent infectious and inflammatory diseases.

## Abstract (precis)

DNA viruses, including hepatitis B virus and herpes simplex virus, induce a series of immune responses in the host and lead to human public health concerns worldwide. In addition to cytokines in the cytoplasm, restriction of viral DNA in the nucleus is an important approach of host immunity. However, the mechanism of foreign DNA recognition and restriction in the cell nucleus is largely unknown. This work demonstrates that an important cellular factor (PJA1) suppresses DNA viruses and transfected plasmids independent of type I and II interferon (IFN) pathways. Instead, PJA1 interacts with the chromosome maintenance complex (SMC5/6), facilitates the complex to recognize and bind viral and episomal DNAs, and recruits DNA topoisomerases to restrict the foreign molecules. These results reveal a distinct mechanism underlying the silencing of viral and episomal invaders in the cell nuclei and suggest that PJA1 acts as a potential agent to prevent infectious and inflammatory diseases.

## INTRODUCTION

Infections by DNA viruses, including hepatitis B virus (HBV) and herpes simplex virus 1 (HSV-1), lead to human public health concerns worldwide. HBV is the leading cause of chronic hepatitis B (CHB), liver cirrhosis (LC), and hepatocellular carcinoma (HCC) (1). The viral genome is a partially double-stranded relaxed circular DNA (rcDNA) replicating by the viral reverse transcriptase from an RNA intermediate (2). HSV-1 is a double-stranded DNA (dsDNA) virus and one of the most common human pathogens, infecting 70 to 90% of the population (3). It may cause diseases ranging from mild conditions to mucocutaneous lesions in lips and skin, herpes keratitis, and herpes encephalitis (4). DNA viruses deliver their genomes into the cell nucleus for transcription and replication. Viral transcription exploits cellular factors but is also repressed by nuclear defense proteins.

PJA1 (Praja1 or RNF70) is a RING-H2 domain E3 ubiquitin ligase expressed mainly in brain, liver, kidney, and embryo (5, 6) and related to liver development and nervous system function (7). Deletion of PJA1 was observed in patients with craniofrontonasal syndrome and associated with mild learning disabilities (8), whereas overexpression of PJA1 facilitates skeletal myogenesis and contributes to neural precursor development (9, 10). Several targets of PJA1-mediated polyubiquitination were identified, including Dlxin-1, Smad3, and PRC2 (11–13). However, the role of PJA1 in the regulation of viral and extrachromosomal DNAs has not been reported.

Structural maintenance of chromosome 5/6 (SMC5/6) is an evolutionarily conserved protein complex (14, 15) that consists of SMC5 and SMC6 along with the NSE1, -2, -3, and -4 proteins (16–18) and plays irreplaceable roles in DNA repair, chromosome segregation, and telomere length maintenance (19–22). Interestingly, in addition to its essential role in chromosome maintenance, the SMC5/6 complex was identified as a host restriction factor against HBV (23–25), but the detailed mechanism underlying the function of the complex in silencing DNA viruses and episomal plasmids remains unknown.

DNA topoisomerases (Tops) are highly conserved enzymes that control DNA supercoiling through transiently breaking and rejoining DNA strands and thus play central roles in maintaining DNA integrity for many vital cellular processes (26–28). Topological entrapment of DNA is essential for the function of SMC family complexes, which have been reported to bind DNA molecules and stimulate Top2-dependent catenation of plasmids (29). Functional cooperation between the SMC5/6 complex and Top1 was suggested for the maintenance of topologically challenged chromosomes (30). More recently, it was reported that Top inhibitors regulate SAMHD1 and human immunodeficiency virus type 1 (HIV-1) permissiveness at a post-reverse-transcription step (31).

This study demonstrates that PJA1 restricts DNA viruses but not RNA viruses and represses transfected reporters but not endogenous or chromosome-integrated genes independent of type I and II interferons (IFNs). Instead, PJA1 interacts with the SMC5/6 complex and diverts the complex to recognize viral and episomal DNAs. Interestingly, Top1 or Top2 is involved in PJA1-mediated repression of episomal DNA. Thus, we reveal a distinct mechanism underlying the restriction of DNA viruses and foreign DNAs in the nucleus, in which PJA1 coordinates with the SMC5/6 complex to silence viral and episomal DNAs in an IFN-independent manner.

## RESULTS

### PJA1 represses the transcription and replication of HBV and HSV-1.

**FIG 1:**
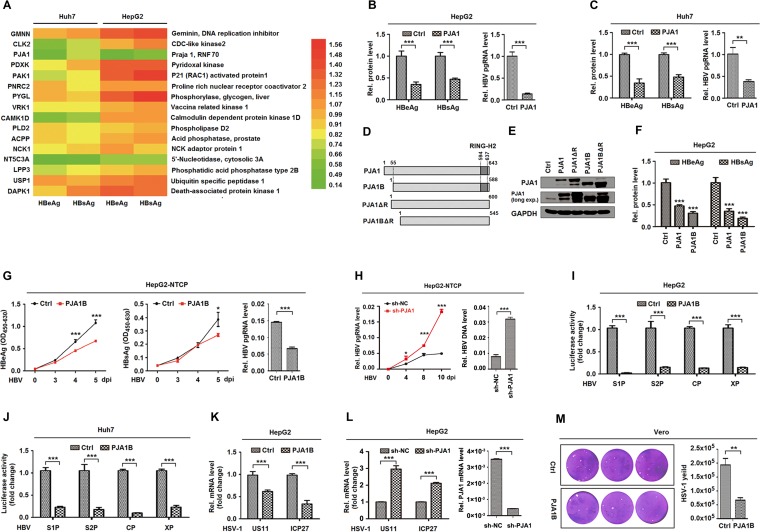
PJA1 represses the transcription and replication of HBV and HSV-1. (A) Huh7 and HepG2 cells were plated in 24-well plates and transfected with 0.2 μg pHBV1.3 and 0.3 μg plasmids expressing 16 candidate proteins in triplicates for 48 h. (B and C) HepG2 cells (B) and Huh7 cells (C) were plated in 24-well plates and transfected with 0.2 μg pHBV1.3 and 0.3 μg pCAGGS-HA-PJA1 in triplicates for 48 h. HBeAg and HBsAg in the supernatants were assayed by an ELISA, and HBV pgRNA was measured by qPCR. (D) Diagrams of PJA1, PJA1B, PJA1ΔR, and PJA1BΔR proteins. (E) 293T cells were plated in 12-well plates and transfected with 1 μg pCAGGS-HA, pCAGGS-HA-PJA1, pCAGGS-HA-PJA1ΔR, pCAGGS-HA-PJA1B, and pCAGGS-HA-PJA1BΔR for 48 h. The expressed proteins were detected by Western blotting using anti-PJA1 antibody. (F) HepG2 cells were plated in 24-well plates overnight and transfected with 0.2 μg pHBV1.3 and 0.3 μg pCAGGS-HA, pCAGGS-HA-PJA1, and pCAGGS-HA-PJA1B for 48 h. HBeAg and HBsAg in the supernatants were assayed by an ELISA. (G) HepG2-NTCP cells were plated in 6-well plates, transfected with 2 μg pCAGGS-HA or pCAGGS-HA-PJA1B, and infected with HBV at 1,000 GE by inoculation with concentrated supernatants of HepaAD38 cells. HBeAg and HBsAg in the supernatants were assayed by an ELISA, and HBV pgRNA was measured by qPCR. OD_450-630_, optical density at 450 to 630 nm. (H) HepG2-NTCP cells were plated in 6-well plates, transfected with 2 μg pLKO.1-sh-NC or -sh-PJA1 for 24 h, and infected with HBV at 1,000 GE by inoculation with concentrated supernatants of HepaAD38 cells. sh-NC, nonspecific control shRNA. HBV pgRNA was measured by qPCR. Total DNA was extracted at 12 days postinfection (dpi), and DNA levels of HBV replication intermediates were measured by qPCR. (I and J) HepG2 cells (I) and Huh7 cells (J) were plated in 24-well plates and transfected with 0.3 μg pCAGGS-HA-PJA1B and 0.2 μg luciferase reporters containing the pre-S1, pre-S2, core, and X promoters of HBV for 48 h. Luciferase activities were determined, and results are expressed as fold induction relative to the control. (K) HepG2 cell lines stably expressing PJA1B were generated and infected with HSV-1 at an MOI of 0.1 for 8 h. HSV-1 *US11* and *ICP27* mRNA levels were determined by RT-qPCR. (L) HepG2-sh-NC and HepG2-sh-PJA1 cells were infected with HSV-1 at an MOI of 0.1 for 8 h. (Left) HSV-1 *US11* and *ICP27* mRNA levels were determined by RT-qPCR. (Right) HepG2 cell lines stably expressing pLKO.1-sh-NC or -sh-PJA1 were generated, and PJA1 mRNA levels in HepG2-sh-NC and HepG2-sh-PJA1 cells were detected. (M) Vero cells were plated in 6-well plates, transfected with 2 μg pCAGGS-HA or pCAGGS-HA-PJA1B for 24 h, and infected with HSV-1 at an MOI of 0.1. At 48 h postinfection, cell culture supernatants were collected, and the viral yields were determined by a plaque assay. Data are shown as means ± SD and correspond to results from a representative experiment out of three performed. **, *P* < 0.01; ***, *P* < 0.001.

We initially evaluated 16 candidate genes that may be involved in the regulation of HBV replication. Enzyme-linked immunosorbent assay (ELISA) results showed that among the candidates, PJA1 significantly represses hepatitis B virus e antigen (HBeAg) and hepatitis B virus s antigen (HBsAg) production in pHBV1.3-transfected cells (Fig. 1A). Further analyses revealed that HBeAg, HBsAg, and HBV pregenomic RNA (pgRNA) were downregulated by PJA1 (Fig. 1B and C), suggesting that PJA1 acts as a potential antiviral factor against HBV. PJA1 encodes two isoforms, PJA1 and PJA1B, with a 55-amino-acid difference (6), and the N-terminus-lacking isoform, PJA1B, was reported to function consistently with full-length PJA1 in nerve growth factor-induced differentiation of rat pheochromocytoma cells (50). Here, we generated four plasmids expressing PJA1; PJA1B; PJA1ΔR, which lacks the RING domain; and PJA1BΔR, which lacks the RING domain (Fig. 1D). The protein products were confirmed, and the detectable endogenous PJA1 protein fits the molecular weight of PJA1B (Fig. 1E). Secreted HBeAg and HBsAg were significantly attenuated by both PJA1 and PJA1B in transfected cells (Fig. 1F), indicating that PJA1 and PJA1B are functionally homologous. In addition, HBeAg, HBsAg, and HBV pgRNA were significantly attenuated by PJA1B in HepG2-NTCP cells (a well-established cell system for HBV infection) transfected with pHA-PJA1B and infected with HBV (Fig. 1G), suggesting that PJA1B overexpression downregulates HBV infection. However, HBV pgRNA and HBV DNA replication intermediates were upregulated in HepG2-NTCP cells treated with short hairpin RNA (shRNA) targeting PJA1 (sh-PJA1) and infected with HBV (Fig. 1H), indicating that PJA1B knockdown upregulates HBV infection. Moreover, the activities of pre-S1, pre-S2, core, and X promoters of HBV were repressed by PJA1B in HepG2 cells (Fig. 1I) and Huh7 cells (Fig. 1J). Thus, we demonstrate that PJA1 represses HBV promoter activation and gene transcription and thereby attenuates HBV replication and infection.

We further determined whether PJA1 has any effect on the replication of HSV-1 containing a liner double-stranded DNA genome. The viral *US11* and *ICP27* mRNAs were significantly attenuated in HepG2 cells stably expressing PJA1B and infected with HSV-1 (Fig. 1K), suggesting that PJA1B overexpression represses HSV-1 gene transcription. However, *US11* and *ICP27* mRNAs were significantly upregulated in HepG2 cells treated with sh-PJA1B and infected with HSV-1 (Fig. 1L), indicating that PJA1B knockdown facilitates HSV-1 gene transcription. Moreover, the viral titer was significantly reduced in the supernatant of Vero cells transfected with pHA-PJA1B and infected with HSV-1 (Fig. 1M), revealing that PJA1B attenuates HSV-1 replication. Taken together, these results demonstrate that PJA1 represses the transcription and replication of the DNA viruses HBV and HSV-1.

### PJA1 represses DNA viruses and episomal plasmids independent of type I and II IFNs.

**FIG 2:**
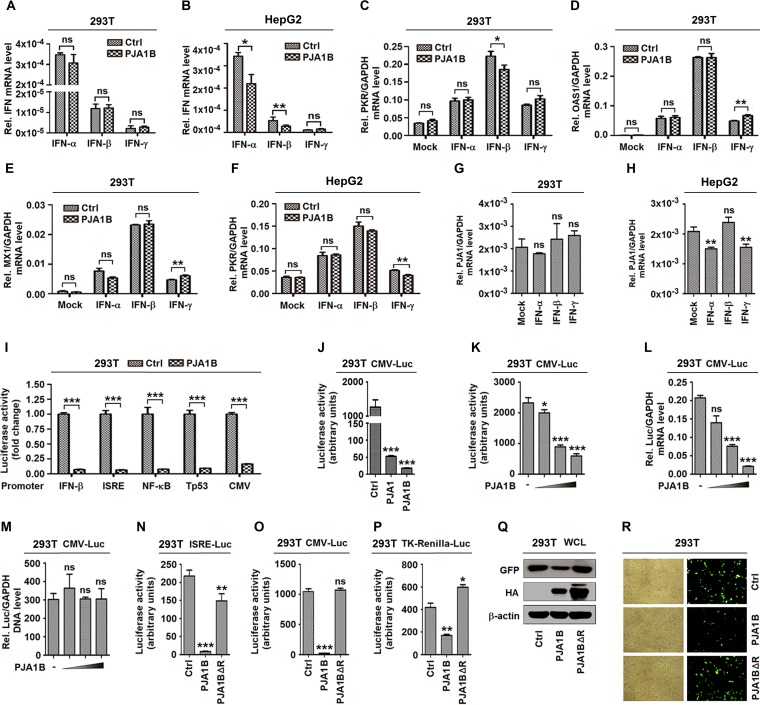
PJA1 generally represses viral and episomal DNAs independent of IFN signaling pathways. (A and B) 293T cells (A) or HepG2 cells (B) were plated in 12-well plates and transfected with 1 μg pCAGGS-HA or pCAGGS-HA-PJA1B for 24 h. The mRNA levels of endogenous *IFN*-α, *IFN*-β, and *IFN*-γ genes were measured by RT-qPCR. (C to E) 293T cells were plated in 12-well plates; transfected with 1 μg pCAGGS-HA or pCAGGS-HA-PJA1B for 24 h; and treated with recombinant human IFN-α (rhIFN-α) (300 IU/ml), rhIFN-β (10 ng/ml), and rhIFN-γ (50 ng/ml) for 12 h. The mRNA levels of endogenous *PKR* (C), *OAS1* (D), and *MX1* (E) genes were measured by RT-qPCR. (F) HepG2 cells were plated in 12-well plates; transfected with 1 μg pCAGGS-HA or pCAGGS-HA-PJA1B for 24 h; and treated with rhIFN-α (300 IU/ml), rhIFN-β (10 ng/ml), and rhIFN-γ (50 ng/ml) for 12 h. The mRNA level of the endogenous PKR gene was measured by RT-qPCR. (G and H) 293T cells (G) or HepG2 cells (H) were treated with rhIFN-α (300 IU/ml), rhIFN-β (10 ng/ml), and rhIFN-γ (50 ng/ml) for 12 h. The *PJA1* and *GAPDH* mRNA levels were measured by RT-qPCR. (I) 293T cells were plated in 24-well plates and cotransfected with 0.3 μg pCAGGS-HA or pCAGGS-HA-PJA1B and 0.2 μg reporters, pIFN-β-Luc, pISRE-Luc, pNF-κB-Luc, pTp53-Luc, and pCMV-Luc for 24 h. Luciferase activities were measured. (J) 293T cells were plated in 24-well plates and cotransfected with 0.3 μg pCAGGS-HA, pCAGGS-HA-PJA1, or pCAGGS-HA-PJA1B and 0.2 μg pCMV-Luc for 24 h. Luciferase activities were measured. (K to M) 293T cells were plated in 24-well plates and cotransfected with 0.2 μg pCMV-Luc and pCAGGS-HA-PJA1B at 0, 0.01, 0.05, and 0.1 μg for 24 h. (K) Luciferase activity was measured. (L) The *Luc* mRNA level was quantified by RT-qPCR. (M) pCMV-Luc DNA in the nuclear extract was quantified by qPCR. (N to P) 293T cells were plated in 24-well plates and cotransfected with 0.3 μg pCAGGS-HA, pCAGGS-HA-PJA1B, or pCAGGS-HA-PJA1BΔR and 0.2 μg reporters, pISRE-Luc (L), pCMV-Luc (M), and pTK-Renilla-Luc (N) for 24 h. Luciferase activities were measured. (Q and R) 293T cells were plated in 12-well plates and transfected with 0.5 μg pEGFP and 0.5 μg pCAGGS-HA, pCAGGS-HA-PJA1B, or pCAGGS-HA-PJA1BΔR for 24 h. (Q) GFP, PJA1, and β-actin were detected by Western blotting. WCL, whole-cell lysate. (R) Fluorescence intensity of EGFP was detected. Data are shown as means ± SD and correspond to results of a representative experiment out of three performed. Results are expressed as fold induction relative to the control. ns, not significant (*P* > 0.05); *, *P* ≤ 0.05; **, *P* < 0.01; ***, *P* < 0.001.

The host immune system utilizes pattern recognition receptors to sense pathogen-associated molecular patterns or damage-associated molecular patterns, leading to immune responses. Viral or cellular DNA has the potential to activate immune responses through different pathways, and the best-characterized one is the activation of interferon regulatory factors (IRFs) and IFNs (32). Since PJA1 attenuates DNA virus replication, we assumed that PJA1 may play a role in the activation of IFN signaling. However, in HEK293T (293T) cells, PJA1B did not induce endogenous type I and II IFN (IFN-α, IFN-β, and IFN-γ) expression (Fig. 2A), while in HepG2 cells, PJA1B slightly attenuated endogenous IFN-α and IFN-β expression and had no effect on IFN-γ expression (Fig. 2B), indicating that PJA1 is not associated with IFN signaling. Similarly, the endogenous interferon-stimulated genes (ISGs) *PKR* (Fig. 2C), *OAS1* (Fig. 2D), and *MX1* (Fig. 2E) induced by recombinant human IFN-α (rhIFN-α), rhIFN-β, and rhIFN-γ were relatively unaffected by PJA1 in 293T cells, revealing that PJA1 is not associated with IFN signaling. Additionally, endogenous *PKR* expression induced by rhIFN-α, rhIFN-β, and rhIFN-γ was relatively unaffected by PJA1 in HepG2 cells (Fig. 2F), confirming that PJA1 is not associated with IFN signaling. Moreover, endogenous *PJA1* mRNA was not induced by rhIFN-α, rhIFN-β, and rhIFN-γ in both 293T cells (Fig. 2G) and HepG2 cells (Fig. 2H), suggesting that type I and II IFNs are not required for PJA1 expression. Thus, we reveal that PJA1 restricts DNA virus replication independent of the IFN pathways but through a different mechanism.

Meanwhile, the roles of PJA1 in regulating different signaling pathways were evaluated. Interestingly, luciferase (Luc) driven by promoters of several irrelevant pathways, including IFN-β–Luc, interferon-stimulated response element (ISRE)–Luc, NF-κB–Luc, Tp53-Luc, and cytomegalovirus (CMV)-Luc, was significantly repressed by PJA1B (Fig. 2I). Basal IFN-β–Luc and ISRE-Luc expressions were repressed by PJA1B, suggesting that inhibition was not through disruption of stimulus-induced recruitment of specific transcription factors. CMV-Luc activity was downregulated by both PJA1 and PJA1B (Fig. 2J), and CMV-Luc activity and mRNA were attenuated by PJA1B in dose-dependent manners (Fig. 2K and L). However, transfected CMV-Luc DNA was not affected by PJA1 (Fig. 2M). These results demonstrate that PJA1B suppresses CMV-Luc mRNA transcription but not CMV-Luc DNA plasmid copy numbers. The RING domain of PJA1 is responsible for its enzyme activity. We demonstrated that the RING domain deletion mutant PJA1BΔR failed to inhibit ISRE-Luc and CMV-Luc (Fig. 2M and O), suggesting that this domain is required for PJA1-mediated episomal plasmid repression. Similarly, HSV-TK Renilla-Luc activity was inhibited by PJA1B and slightly induced by PJA1BΔR (Fig. 2P). Moreover, enhanced green fluorescent protein (EGFP) production (Fig. 2Q) and intensity (Fig. 2R) were attenuated by PJA1B but not by PJA1BΔR. Thus, we demonstrate that PJA1 represses transfected plasmids regardless of the DNA sequence features of their enhancers, promoters, and coding sequences (CDSs).

### PJA1 has no effect on chromosome-integrated genes and RNA viruses.

**FIG 3:**
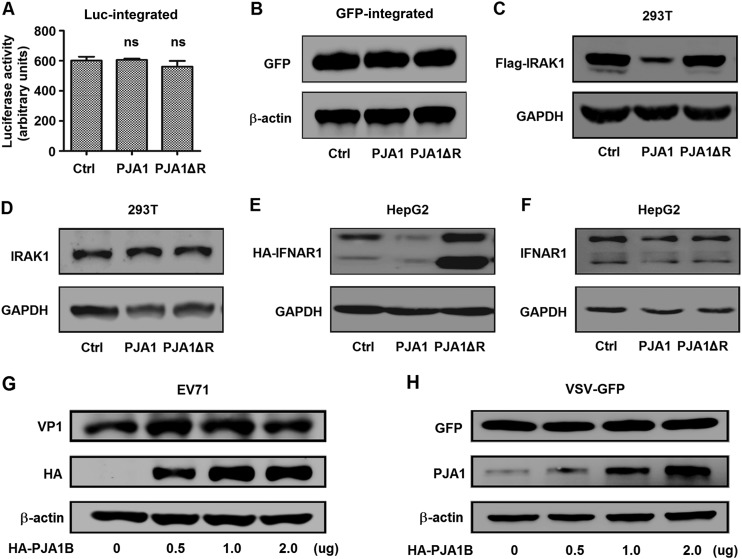
PJA1 has no effect on chromosome-integrated genes and RNA viruses. (A) 293T-Luc cells were generated, in which the CMV promoter driving Luc was randomly integrated into cell chromosomes by a lentiviral system. The cells were plated in 24-well plates and transfected with 0.5 μg pCAGGS, pCAGGS-HA-PJA1, or pCAGGS-HA-PJA1ΔR for 48 h. Luciferase activities were measured. Data are shown as means ± SD and correspond to results of a representative experiment out of three performed. ns, nonsignificant. (B) 293T-EGFP cells were generated, in which the CMV promoter driving EGFP was randomly integrated into cellular chromosomes by a lentiviral system. The cells were plated in 12-well plates and transfected with 1 μg pCAGGS, pCAGGS-HA-PJA1, or pCAGGS-HA-PJA1ΔR. GFP and β-actin protein were detected by Western blot analyses. (C) 293T cells were plated in 12-well plates and cotransfected with 0.5 μg pFlag-IRAK1 and 0.5 μg pCAGGS, pCAGGS-HA-PJA1, or pCAGGS-HA-PJA1ΔR for 48 h. Flag-IRAK1 and GAPDH protein levels were determined by Western blot analyses. (D) 293T cells were plated in 12-well plates and transfected with 1 μg pCAGGS, pCAGGS-HA-PJA1, or pCAGGS-HA-PJA1ΔR for 48 h. Endogenous IRAK1 and GAPDH were detected by Western blot analyses. (E) HepG2 cells were plated in 12-well plates and cotransfected with 0.5 μg pCAGGS-HA-IFNAR1 and 0.5 μg pcDNA3.1, pcDNA3.1-PJA1, or pcDNA3.1-PJA1ΔR for 48 h. HA-IFNAR1 and GAPDH were detected by Western blot analyses. (F) HepG2 cells were plated in 12-well plates and transfected with 1 μg pcDNA3.1, pcDNA3.1-PJA1, or pcDNA3.1-PJA1ΔR for 48 h. Endogenous IFNAR1 and GAPDH were detected by Western blot analyses. (G) RD cells were plated in 6-well plates, transfected with pCAGGS-HA-PJA1B at different concentrations (0, 0.5, 1, and 2 μg) for 48 h, and infected with EV71 at an MOI of 5 for 6 h. EV71 VP1, HA-PJA1B, and GAPDH were detected by Western blot analyses. (H) 293T cells were plated in 6-well plates, transfected with pCAGGS-HA-PJA1B at different concentrations (0, 0.5, 1, and 2 μg) for 24 h, and infected with VSV-GFP at an MOI of 1 for 12 h. GFP, PJA1B, and GAPDH were detected by Western blot analyses.

Since PJA1 restricts DNA viruses and extrachromosomal plasmids independent of IFN pathways, we assumed that PJA1 may recognize extrachromosomal DNAs by their distinct structure features from cell chromosomes. We determined whether PJA1 also represses genome-integrated plasmids and RNA viruses. Initially, we generated two stable cell lines, 293T-Luc and 293T-EGFP, in which the CMV promoter driving the *Luc* gene or the *EGFP* gene was integrated into cell chromosomes by a lentiviral system. 293T was chosen for maximum efficiency of the following transfections. Luciferase activity in 293T-Luc stable cells and EGFP production in 293T-GFP stable cells were not affected by PJA1 or PJA1ΔR (Fig. 3A and B). Interestingly, plasmid-expressed interleukin 1 receptor-associated kinase 1 (IRAK1) was significantly attenuated by PJA1 and not by PJA1ΔR, but endogenous IRAK1 was not affected by PJA1 or PJA1ΔR (Fig. 3C and D). Similarly, plasmid-expressed interferon alpha and beta receptor subunit 1 (IFNAR1) was significantly downregulated by PJA1 and not by PJA1ΔR, but endogenous IFNAR1 was not affected by PJA1 or PJA1ΔR (Fig. 3E and F).

We then investigated the role of PJA1 in the replication of the RNA viruses enterovirus 71 (EV71), containing a single-stranded and positive-sense RNA genome, and vesicular stomatitis virus (VSV), carrying a single-stranded and negative-sense RNA genome. The EV71 VP1 protein was not affected by PJA1B in rhabdomyosarcoma (RD) cells transfected with pHA-PJA1B and infected with EV71 (Fig. 3G), revealing that PJA1 has no effect on EV71 replication. Green fluorescent protein (GFP) was relatively unchanged by PJA1 in 293T cells transfected with pHA-PJA1B and infected with a recombinant VSV (VSV-GFP) (Fig. 3H), suggesting that PJA1 has no effect on VSV replication. Taken together, these results demonstrate that PJA1 restricts DNA viruses and transfected plasmids but not chromosome-integrated plasmids, endogenous genes, or RNA viruses.

### Knockout of PJA1 facilitates the transcription of ectopic DNA.

**FIG 4:**
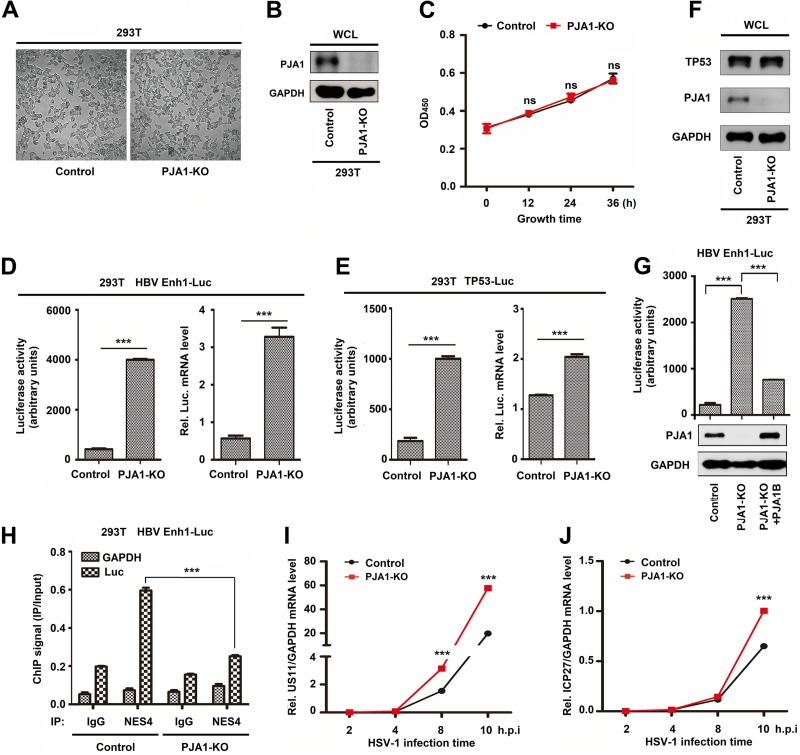
Knockout of PJA1 facilitates transcription of ectopic DNA. (A and B) To measure the effect of endogenous PJA1 on the regulation of episomal gene activation and DNA virus replication, the Cas9-mediated PJA1 knockout cell line 293T(PJA1-KO) and the control cell line with the pSpCas9-2A-Puro-MCS vector integrated were generated. Shown are bright-field images (A) and PJA1 protein levels (B) for 293T control cells and 293T(PJA1-KO) cells. (C) 293T control cells and 293T(PJA1-KO) cells were plated in a 96-well plate at a density of 1,000 cells/well in 100 μl of culture medium in triplicates. Ten microliters of a CCK-8 solution was added to each well of the plate, and the plate was incubated for 2 h in an incubator. The absorbance was measured at 450 nm. (D and E) 293T control cells and 293T(PJA1-KO) cells were plated in 24-well plates in triplicate and then transfected with 0.2 μg HBV Enh1-Luc (D) and 0.2 μg Tp53-Luc (E) for 24 h, and luciferase activity and relative luciferase mRNA levels were measured. (F) Tp53 protein levels in 293T control cells or 293T(PJA1-KO) cells were detected by Western blot analysis. (G) 293T control cells and 293T(PJA1-KO) cells were plated in 12-well plates and transfected with 0.2 μg pHBV-Enh1-Luc with or without 2 ng pCAGGS-HA-PJA1B for 24 h. Luciferase activities of HBV Enh1-Luc and protein levels of PJA1 and GAPDH were measured. (H) 293T control cells and 293T(PJA1-KO) cells were plated in 6-cm dishes and transfected with 5 μg pHBV-Enh1-Luc for 24 h. NSE4 binding to the episomal reporter or the GAPDH gene was monitored by ChIP assays using anti-NSE4 antibody. The amount of immunoprecipitated DNA was determined by RT-qPCR. (I and J) 293T control cells and 293T(PJA1-KO) cells were plated in 12-well plates overnight and then infected with HSV-1 at an MOI of 0.1 for the indicated times. HSV-1 *US11* gene mRNA (I) and *ICP27* gene mRNA (J) levels were determined by RT-qPCR. h.p.i, hours postinfection. Data are shown as means ± SD and correspond to results of a representative experiment out of three performed. ***, *P* < 0.001.

To measure the effect of endogenous PJA1 on the regulation of episomal gene activation and DNA virus replication, a Cas9-mediated PJA1 knockout (PJA1-KO) cell line, 293T(PJA1-KO), and a control cell line (293T with the pSpCas9-2A-Puro-MCS vector integrated) were generated. Morphologically, 293T(PJA1-KO) cells generally resemble 293T control cells (Fig. 4A). PJA1 protein was expressed in 293T control cells but was not detected in 293T(PJA1-KO) cells (Fig. 4B), indicating that PJA1 was successfully knocked out in the PJA1-KO cell line. The effect of PJA1-KO on cell proliferation was evaluated by cell counting kit 8 (CCK8) assays. The proliferation of 293T(PJA1-KO) cells had no significant difference compared to that of 293T control cells (Fig. 4C), revealing that PJA1 knockout does not affect cell proliferation. 293T control cells and 293T(PJA1-KO) cells were transfected with pHBV-Enhancer 1 (Enh1)-Luc and pTp53-Luc for 24 h, respectively. The activities and mRNA levels of luciferase driven by HBV Enh1 were significantly higher in 293T(PJA1-KO) cells than in 293T control cells (Fig. 4D). Similarly, the activities and mRNA levels of luciferase driven by Tp53 transcriptional recognition sequences were significantly higher in 293T(PJA1-KO) cells than in 293T control cells (Fig. 4E). Endogenous Tp53 protein was not affected in both 293T(PJA1-KO) cells and 293T control cells (Fig. 4F), indicating that knockout of PJA1 has no effect on Tp53 expression. Luciferase activities driven by HBV Enh1 were activated in 293T(PJA1-KO) cells transfected with HBV Enh1-Luc but significantly attenuated by PJA1B in 293T(PJA1-KO) cells cotransfected with HBV Enh1-Luc and pHA-PJA1B (Fig. 4G), demonstrating that PJA1 represses HBV Enh1 activation. Additionally, 293T control cells and 293T(PJA1-KO) cells were transfected with pHBV-Enh1-Luc. The binding of NSE4 with episomal Enh1-Luc DNA or glyceraldehyde-3-phosphate dehydrogenase (GAPDH) DNA as a negative control was monitored by chromatin immunoprecipitation (ChIP) assays. The binding of NSE4 to Enh1-Luc DNA was significantly reduced in 293T(PJA1-KO) cells compared to that in 293T control cells (Fig. 4H), indicating that PJA1 facilitates the binding of NSE4 to Enh1-Luc. Moreover, 293T control cells and 293T(PJA1-KO) cells were infected with HSV-1. The levels of HSV-1 *US11* mRNA (Fig. 4I) and *ICP27* mRNA (Fig. 4J) were significantly higher in 293T(PJA1-KO) cells than in 293T control cells at 10 h postinfection, suggesting that PJA1 knockout upregulates HSV-1 gene transcription. Taken together, these data reveal that PJA1 represses the transcription of ectopic plasmids and DNA viruses.

### PJA1 interacts with the SMC5/6 complex in the nucleus.

**FIG 5:**
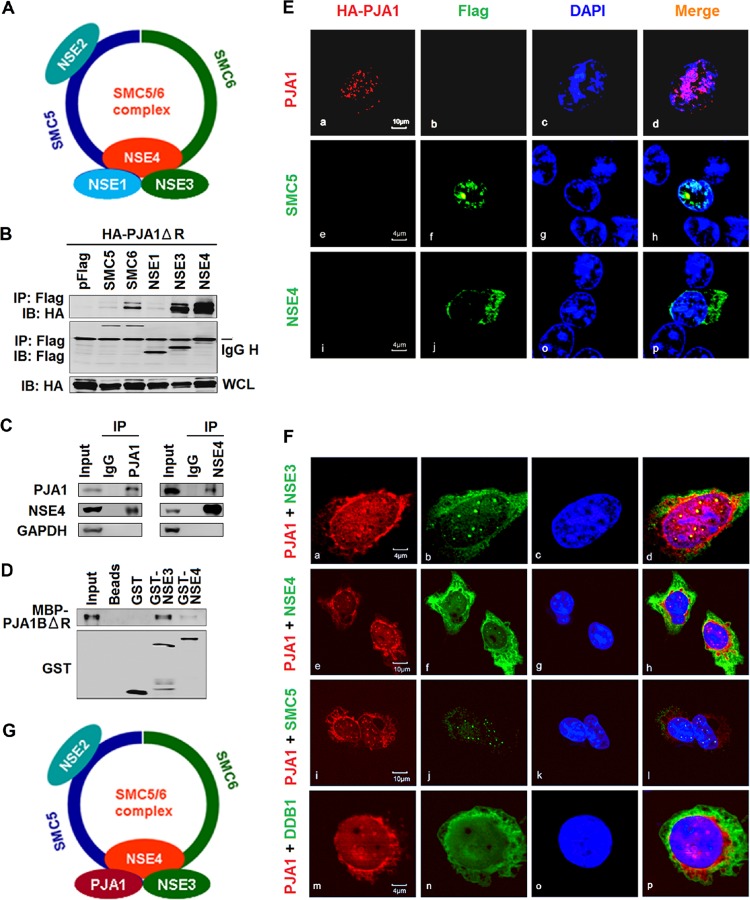
PJA1 interacts with the SMC5/6 complex in the nucleus. (A) Schematic of the SMC5/6 complex. (B) 293T cells were plated in 6-well plates and cotransfected with 1 μg pCAGGS-HA-PJA1ΔR and 1 μg pFlag, pFlag-SMC5, pFlag-SMC6, pFlag-NSE1, pFlag-NSE3, or pFlag-NSE4. Cells were lysed in RIPA lysis buffer. The immunoprecipitates and whole-cell lysates were analyzed by Western blotting with anti-HA or anti-Flag antibody. IB, immunoblot. (C) 293T cells were lysed in RIPA lysis buffer, and cell lysates were immunoprecipitated with anti-PJA1 antibody, anti-NSE4 antibody, or rabbit IgG. The immunoprecipitates and whole-cell lysates were analyzed by Western blotting with antibody to PJA1, NSE4, or GAPDH. (D) GST pulldown with cell lysates containing MBP-PJA1BΔR and GST, GST-NSE3, or GST-NSE4 expressed and purified from E. coli. After pulldown, precipitates were analyzed by Western blotting with anti-PJA1 or anti-GST antibody. (E and F) HepG2 cells were plated in confocal dishes and transfected with 1 μg pCAGGS-HA-PJA1, pFlag-SMC5, or pFlag-NSE4 (E) or cotransfected with 1 μg pCAGGS-HA-PJA1 and 1 μg pFlag-SMC5, pFlag-NSE3, pFlag-NSE4, or pFlag-DDB1 (F). Cells were immunostained with anti-HA and anti-Flag antibodies. Immunofluorescence analysis shows PJA1 (red), NSE3/4 (green), SMC5 (green), and DDB1 (green). The nucleus was stained by DAPI. (G) Schematic of the SMC5/6 complex in which NSE1 was replaced by PJA1.

The mechanism by which PJA1 restricts DNA viruses and episomal plasmids was investigated. Based on a large interactome screening, it was predicted that PJA1 may interact with the SMC5/6 complex (33), which consists of SMC5 and SMC6 along with the NSE1, -2, -3, and -4 proteins (Fig. 5A). Since knockdown of SMC5/6 rescues the replication of HBx-deficient HBV and enhances the expression of the transfected episomal HBV Enh1-driven firefly luciferase reporter as well as the episomal Renilla luciferase construct driven by the CMV promoter (23), we speculated that PJA1 may silence extrachromosomal DNA through interacting with the complex. Coimmunoprecipitation (co-IP) results showed that PJA1ΔR (the RING domain deletion mutant was used to avoid inhibition of cotransfected plasmids by PJA1) was coprecipitated with SMC6, NSE3, and NSE4 but not with NSE1 (Fig. 5B). Endogenous co-IP confirmed that endogenous PJA1 interacted with endogenous NSE4 (Fig. 5C, left) and that NSE4 interacted with endogenous PJA1 (Fig. 5C, right). A glutathione *S*-transferase (GST) pulldown assay using fusion proteins overexpressed and purified from Escherichia coli revealed that maltose-binding protein (MBP)–PJA1BΔR strongly interacted with GST-NSE3 and weakly interacted with GST-NSE4 (Fig. 5D). The distribution and association of PJA1 with the components of the SMC5/6 complex were examined by confocal microscopy. In the presence of a single protein, PJA1 was located in the nucleus (Fig. 5Ea to d), SMC5 was mainly distributed in the nucleus (Fig. 5Ee to h), and NSE4 was present in both the cytoplasm and nucleus (Fig. 5Ei to p). Interestingly, in the presence of two proteins, PJA1 colocalized with NSE3 (Fig. 5Fa to d), NSE4 (Fig. 5Fe to h), and SMC5 (Fig. 5Fi to l) to form distinctive spots in the nucleus but was not colocalized with an unrelated protein, DDB1 (Fig. 5Fm to p). Taken together, our data reveal that PJA1 interacts with the SMC5/6 complex in the nucleus independent of its RING domain (Fig. 5G).

### PJA1 facilitates the binding of the SMC5/6 complex with viral and episomal DNAs.

**FIG 6:**
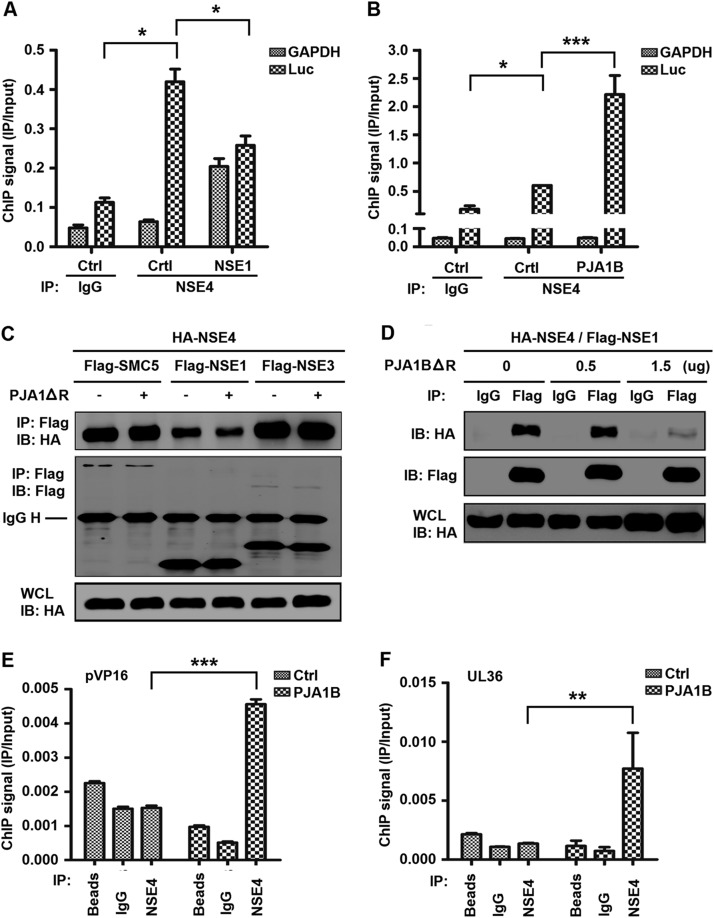
PJA1 facilitates the binding of the SMC5/6 complex to viral and episomal DNAs. (A and B) 293T cells were plated in 6-cm dishes and cotransfected with 3 μg CMV-Luc and then with 2 μg pFlag-NSE1 (A) or 2 μg pcDNA3.1-PJA1B (B). NSE4 binding of the episomal reporter or *GAPDH* gene was monitored by ChIP assays using anti-NSE4 antibody. The level of immunoprecipitated DNA was determined by RT-qPCR. (C) 293T cells were plated in 6-well plates and cotransfected with 0.5 μg pCAGGS-HA-NSE4 and 0.5 μg pFlag-SMC5, pFlag-NSE1, or pFlag-NSE3 with or without 0.5 μg pcDNA3.1-PJA1ΔR. (D) 293T cells were plated in 6-well plates and cotransfected with 0.5 μg pCAGGS-HA-NSE4 and 0.5 μg pFlag-NSE1 and then with pcDNA3.1-PJA1BΔR at different concentrations (0, 0.5, and 1.5 μg). Cells were lysed in RIPA lysis buffer. The immunoprecipitates and whole-cell lysates were analyzed by Western blotting with anti-HA or anti-Flag antibody. (E and F) HepG2 cells stably expressing PJA1B were plated in 6-cm dishes and infected with HSV-1 at an MOI of 10 for 8 h. The binding of NSE4 to the *VP16* gene promoter (E) or *UL36* gene (F) of HSV-1 was monitored by a ChIP assay using beads only, rabbit IgG, or anti-NSE4 antibody. Data are shown as means ± SD and correspond to results of a representative experiment out of three performed. *, *P* < 0.05; **, *P* < 0.01; ***, *P* < 0.001.

Since PJA1 interacts with the SMC5/6 complex, we determined whether PJA1 cooperated with the complex in restricting viral and extrachromosomal DNAs. The SMC5/6 ring structure entraps DNA, and the NSE1/3/4 subcomplex binds to dsDNA and loads DNA onto the ring (34). Here, ChIP assays revealed that the interaction of NSE4 with CMV-Luc DNA was not affected by NSE1 (a SMC5/6 complex protein containing a RING domain analogous to that of PJA1) (35) but was significantly enhanced by PJA1B (Fig. 6A and B). In addition, PJA1ΔR attenuated the interaction of NSE4 with NSE1 but not interactions of NSE4 with SMC5 or NSE3 and downregulated the interaction of NSE1 with NSE4 in a dose-dependent manner (Fig. 6C and D), revealing that PJA1 may compete with NSE1 in interacting with the SMC5/6 complex. Moreover, PJA1B significantly enhanced the binding of the SMC5/6 complex to HSV-1 *VP16* promoter DNA and *UL36* gene DNA in HepG2 cells stably expressing PJA1B and infected with HSV-1 (Fig. 6E and F), demonstrating that PJA1 promotes the binding of the SMC5/6 complex to HSV-1 genomic DNA. Therefore, we reveal that PJA1 collaborates with the SMC5/6 complex in binding viral and extrachromosomal DNAs.

### DNA topoisomerases are involved in PJA1-mediated restriction of viral and episomal DNA.

**FIG 7:**
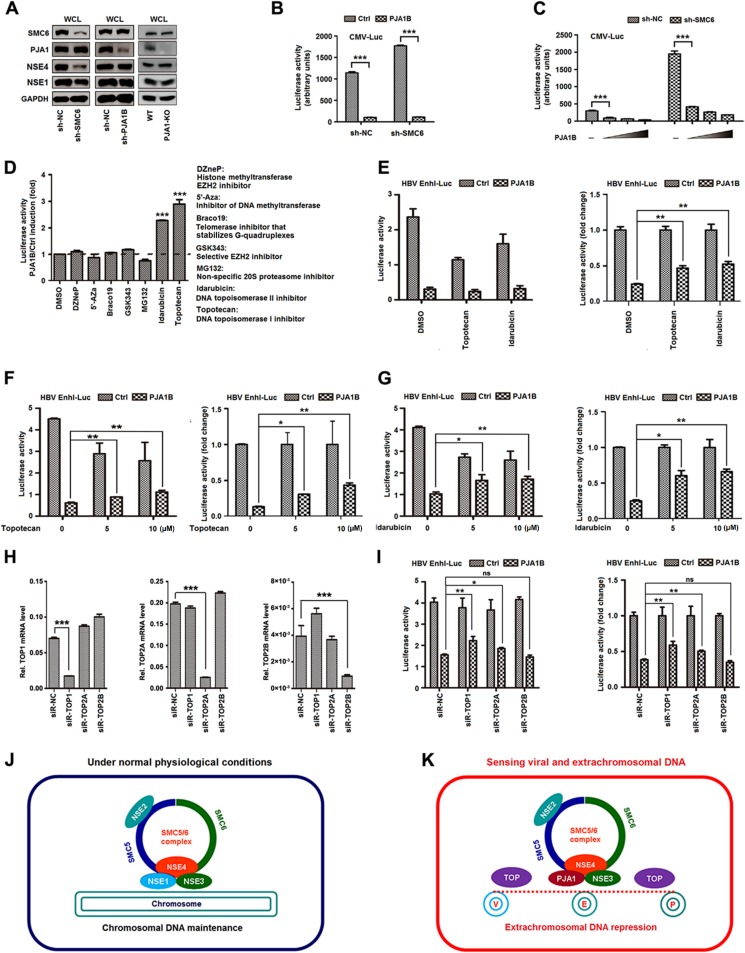
DNA topoisomerases are involved in PJA1-mediated restriction of viral and episomal DNAs. (A) 293T cells were plated in 12-well plates and transfected with 1 μg sh-NC, sh-SMC6, or sh-PJA1 for 48 h. SMC6, PJA1, NSE4, NSE1, and GAPDH proteins expressed in the transfected cells and in 293T control or 293T(PJA1-KO) cells were detected by Western blot analyses using the corresponding antibodies. WT, wild type. (B) 293T cells were plated in 24-well plates, cotransfected with 0.2 μg pCMV-Luc and 0.2 μg sh-SMC6 or sh-NC for 48 h, and then transfected with 0.2 μg pCAGGS or pCAGGS-HA-PJA1B for 48 h. Luciferase activities were measured. (C) Two stable cell lines were generated, in which sh-NC and sh-SMC6 were stably expressed in HepG2 cells. The stable cells were cotransfected with 0.1 μg pCMV-Luc and pCAGGS-HA-PJA1B at different concentrations (0, 0.02, 0.05, and 0.1 μg) for 48 h. Luciferase activities were measured. (D to G) 293T cells were plated in 24-well plates and cotransfected with 0.3 μg pCAGGS-PJA1B and 0.2 μg pHBV-Enh1-Luc for 24 h. The transfected cells were treated with different inhibitors (D); with topotecan or idarubicin at 5 μM for 10 h (E); or with topotecan (F) or idarubicin (G) at concentrations of 1, 5, and 10 μM for 10 h. Luciferase activity was measured, and results are expressed as primary data (left) and fold induction relative to the control (right). (H) 293T cells were plated in 24-well plates and transfected with 150 μM siR-NC, siR-Top1, siR-Top2a, or siR-Top2b for 48 h. The *Top1* (left), *Top2a* (middle), *Top2b* (right), and *GAPDH* mRNA levels were determined by RT-qPCR, and results are expressed as fold induction relative to the control. (I) 293T cells were plated in 24-well plates; cotransfected with 0.1 μg pEnh1-Luc and 150 μM siR-NC, siR-Top1, siR-Top2a, or siR-Top2b for 24 h; and then transfected with 0.2 μg pCAGGS or pCAGGS-HA-PJA1B for 24 h. Luciferase activity was measured, and results are expressed as primary data (left) and fold induction relative to the control (right). Data are shown as means ± SD and correspond to results of a representative experiment out of three performed. ns, nonsignificant; *, *P* < 0.05; **, *P* < 0.01; ***, *P* < 0.001. (J and K) Diagrams of proposed models for PJA1 function. (J) Under normal conditions, the NSE1/NSE3/NSE4 subcomplex ensures that the SMC5/6 complex maintains the host chromosome. (K) In response to viral and episomal DNAs, PJA1 replaces NSE1 to form the PJA1/NSE3/NSE4 subcomplex, which converts the function of the SMC5/6 complex to the restriction of viral (V), extrachromosomal (E), and plasmid (P) DNA molecules.

The functional correlation between PJA1 and the SMC5/6 complex was further verified. Plasmids expressing shRNAs targeting SMC6 (sh-SMC6) and PJA1B (sh-PJA1B) were generated and then transfected into 293T cells. Results showed that sh-SMC6 significantly attenuated SMC6 and NSE4 protein levels, slightly reduced NSE1 production, and had no effect on PJA1 and GAPDH proteins (Fig. 7A, left), which was consistent with a previous report showing that knockdown of any protein of the SMC5/6 complex leads to degradation of other components and disruption of complex formation (36). In addition, sh-PJA1B significantly attenuated PJA1 production but not SMC6, NSE4, NSE1, or GAPDH expression (Fig. 7A, middle), suggesting that knockdown of PJA1 has no effect on the production of SMC5/6 complex components. Moreover, the productions of SMC6, NSE4, and NSE1 proteins were not affected in 293T(PJA1-KO) cells compared to those in 293T cells (Fig. 7A, right), confirming that knockout of PJA1 has no effect on the production of SMC5/6 complex components. 293T cells were cotransfected with pCMV-Luc, sh-SMC6, and pHA-PJA1B. Luciferase activity was repressed by PJA1B in the presence or absence of sh-SMC6 (Fig. 7B), indicating that knockdown of SMC6 has no effect on PJA1-mediated repression of CMV-Luc. To confirm this observation, we generated two stable cell lines in which sh-NC and sh-SMC6 were stably expressed in HepG2 cells. The stable cells were cotransfected with pCMV-Luc and/or pHA-PJA1B. CMV-Luc activity was significantly higher in the presence of sh-SMC6 than that in the absence of sh-SMC6 (Fig. 7C), indicating that knockdown of SMC6 upregulates CMV-Luc. Interestingly, CMV-Luc activity was significantly reduced by PJA1B in the presence or absence of sh-SMC6 (Fig. 7C), suggesting that knockdown of SMC6 has no effect on PJA1-mediated repression of CMV-Luc. Thus, these results suggest that PJA1 functions downstream of the SMC5/6 complex.

The mechanism by which PJA1 represses episomal plasmids was evaluated. Initially, we determined the effects of 8 potential inhibitors on PJA1B-mediated repression of the HBV Enh1-Luc reporter. Among the inhibitors, topotecan (a specific inhibitor of topoisomerase 1) and idarubicin (a specific inhibitor of topoisomerase 2) significantly suppressed PJA1B-mediated repression of HBV Enh1-Luc activity (Fig. 7D), indicating that Top1 and Top2 participated in the repression of HBV Enh1-Luc activity mediated by PJA1. The results confirmed that topotecan and idarubicin recovered PJA1B-mediated repression of the HBV Enh1-Luc reporter in dose-dependent fashions (Fig. 7E to G).

To further confirm the role of topoisomerases in PJA1-mediated repression of extrachromosomal and viral DNAs, we constructed small interfering RNAs (siRNAs) targeting Top1 (siR-Top1), Top2a (siR-Top2a), and Top2b (siR-Top2b). The specificity and efficiency of the siRNAs were verified and confirmed in 293T cells (Fig. 7H). Like the inhibitors, siR-Top1 and siR-Top2a significantly attenuated PJA1B-mediated repression of HBV Enh1-Luc (Fig. 7I). These results demonstrate that DNA topoisomerases are involved in the restriction of viral and extrachromosomal DNAs mediated by PJA1. Therefore, we propose that PJA1 coordinates with the SMC5/6 complex to restrict viral and episomal DNAs through topoisomerases (Fig. 7J and K).

## DISCUSSION

Multiple DNA sensors and adaptors detecting pathogen-derived nucleic acids in the cytoplasm have been identified, including the cytosolic DNA sensor DAI (ZBP1), AIM2, and cGAS (32, 37, 38). The molecular mechanism by which host cells sense microbial and episomal DNAs to eliminate foreign invaders in the nucleus remains largely unknown. One primary concern is how cells distinguish the viral genome from the host genome in the same compartment. This study reveals a distinct mechanism underlying the silencing of foreign DNA in the nucleus mediated by PJA1. PJA1 restricts DNA viruses and extrachromosomal DNA but not RNA viruses or chromosome-integrated plasmids. Notably, PJA1 has no effect on the production of type I and II IFNs and antiviral proteins, and IFNs have no effect on the expression of PJA1, demonstrating that PJA1 restricts foreign DNA independent of type I and II IFN pathways but through different mechanisms.

Interestingly, we reveal that PJA1 plays an important role in the restriction of DNA viruses and extrachromosomal DNAs coordinating with the SMC5/6 complex, which was identified previously as a host restriction factor against HBV (23–25). Additionally, a recent report has shown that SMC5/6 restricts HBV infection in primary human hepatocytes (PHHs) without inducing an IFN response (39). We further demonstrate that PJA1 interacts with the SMC5/6 complex in the nucleus and facilitates the binding of NSE4 to viral and episomal DNAs. The interaction of NSE4 with episomal DNA is enhanced by PJA1 but not by NSE1, whereas PJA1 attenuates the interaction of NSE4 with NSE1. These results potentially suggest that PJA1 competes with NSE1 in interacting with the SMC5/6 complex and may replace NSE1 to facilitate the interaction of NSE4 with episomal DNA. Both PJA1 and NSE1 share a RING domain that commonly indicates E3 ubiquitin ligase activity (11, 12). The RING domain of NSE1 supports the SMC5/6 complex in genome stability maintenance, and our work reveals that the RING domain is required for PJA1 in the restriction of viral and plasmid DNAs. Multiple RING domain proteins were identified as being binding partners of melanoma-associated antigen (MAGE) proteins (40–42). It is reasonable to speculate that similarly to how NSE1 interacts with NSE4 to form the NSE1 (RING)/NSE4 (MAGE) subcomplex, PJA1 may also interact with NSE4 to form a PJA1 (RING)/NSE4 (MAGE) subcomplex to establish specific functions. Thus, we propose that NSE1/3/4 ensures the normal function of the SMC5/6 complex in host chromosome maintenance, whereas PJA1/NSE3/4 converts the function of the SMC5/6 complex to viral DNA restriction, although the underlying physiological significance of the alternative RING-MAGE heterodimers needs to be further investigated.

More interestingly, treatment with inhibitors of DNA topoisomerases and knockdown of the enzymes result in attenuation of PJA1-mediated restriction of HBV Enh1-Luc, demonstrating an important role of Tops in this process. The SMC5/6 complex is loaded onto chromosomes at collapsed replication forks and at sites of DNA damage where chromosome DNA shows abnormal structures and possibly binds through topological entrapment of DNA strands (43, 44). Thus, we suggest that instead of the IFN pathways, PJA1 coordinates with the SMC5/6 complex to recognize viral and episomal DNA structures and recruit DNA Tops to restrict the foreign DNA molecules. This work reveals a distinct mechanism of silencing of foreign DNA invaders in the nuclei and suggests that PJA1 may act as a potential agent for the prevention and treatment of infectious and inflammatory diseases.

## MATERIALS AND METHODS

### Cell cultures and generation of stable cell lines.

African green monkey kidney (Vero) cells and human hepatoma (HepG2) cells were obtained from the American Type Culture Collection (ATCC) (Manassas, VA, USA). Human hepatocarcinoma (Huh7) cells, human embryonic kidney (HEK293T) cells, and human rhabdomyosarcoma (RD) cells were obtained from the China Center for Type Culture Collection (CCTCC) (Wuhan, China). Cells were grown in Dulbecco's modified Eagle medium (DMEM) (Gibco, Grand Island, NY, USA) supplemented with 10% fetal bovine serum (FBS) (Gibco), 100 U/ml penicillin, and 100 μg/ml streptomycin sulfate at 37°C in 5% CO_2_. Cells were transfected with Lipofectamine 2000 (Invitrogen, IL, USA) according to the manufacturer's instructions.

To generate the stable cell lines HepG2-3Flag, HepG2-PJA1B, 293T-Luc, 293T-GFP, HepG2-sh-NC, HepG2-sh-PJA1, and HepG2-sh-SMC6, lentiviruses carrying the encoding gene or target interfering shRNA sequences were produced by cotransfecting the corresponding constructs, the psPAX2 and pMD2.G plasmids, into HEK293T cells. Cells stably expressing the genes of interest were selected with 2.5 μg/ml puromycin for a week. The protein level for the target gene was verified by Western blotting. (Note that the titer of PJA1B-overexpressing lentiviral particles was generally dozens of times lower than that of control lentiviral particles.)

### Generation of PJA1 knockout cells.

PJA1 knockout (PJA1-KO) cells were generated from HEK293T cells with the CRISPR-Cas9-2hitKO system according to the manufacturer's instructions (Beijing Pitoop Bioscientific Inc., Beijing, China). Target guide RNAs (target 1, 5′-CACCGTCTCCCCTGCCACATCGGTT-3′; target 2, 5′-CACCGTTCCACTACTCGTCGTAGTT-3′) were predicted online (Atum). 293T cells (5 × 10^5^) were transfected with 2 μg of a CRISPR-Cas9 vector carrying two guide RNA-expressing cassettes in six-well tissue culture plates for 2 days. The cells were transferred to 10-cm dishes and cultured with medium containing puromycin (1.5 μg/ml) for a week. The culture medium was changed every 2 to 3 days. Individual cell colonies were isolated by limiting dilution. After 1 to 2 weeks, the cells were observed under a microscope, and cells from those wells containing only one cell colony were selected and allowed to expand from a 96-well plate to a 6-well plate. Knockout efficiency was assessed by Western blotting and verified by genomic DNA sequencing.

### Viruses and infection.

For HBV infection, the HBV inoculum was concentrated 100-fold from the supernatants of HepaAD38 cells (provided by Ying Zhu of Wuhan University, China) by ultracentrifugation at 100,000 × *g* for 5 h at 4°C. For infection, HepG2-NTCP cells (provided by Ying Zhu, Wuhan University, China) were plated on collagen I-coated plates in DMEM overnight, and the medium was then changed to PMM (Williams' E medium supplemented with insulin, transferrin, and sodium selenite (ITS), 2 mM l-glutamine, 10 ng/ml of human epidermal growth factor [EGF], 18 μg/ml of hydrocortisone, 40 ng/ml of dexamethasone, 2% dimethyl sulfoxide [DMSO], 100 U/ml of penicillin, and 100 μg/ml of streptomycin) for 6 h. Cells were then infected with 1,000 genome equivalents (GE) per cell of HBV in PMM containing 4% (wt/vol) polyethylene glycol 8000 (PEG 8000) for 16 h. Virus-containing medium was then removed, and the cells were washed five times and further incubated in PMM with 2% serum (45).

HSV-1 strain F (provided by Ying Zhu, Wuhan University, China) was propagated in Vero cells as previously described (46). For infection, cells were exposed to HSV-1 at the indicated multiplicity of infection (MOI) for 1 h at 37°C, washed twice with a phosphate-buffered saline (PBS) solution, and overlaid with fresh DMEM containing 10% FBS. Infected cells were incubated at 37°C for the indicated lengths of time. HSV-1 progeny were titrated on Vero cells as previously described (46). In brief, Vero cells were plated in six-well plates to high confluence (>80%), exposed to HSV-1 for 1 h, and maintained in DMEM-agarose for 72 h. The cells were fixed with 4% (wt/vol) paraformaldehyde for 30 min, washed three times with PBS, and stained with crystal violet dyes.

The recombinant enhanced green fluorescent protein (EGFP)-expressing vesicular stomatitis virus strain (VSV-GFP) (provided by Mingzhou Chen of Wuhan University) was amplified in Vero cells, and the titer was determined on Vero cells by fluorescence microscopy. Cells were incubated with VSV-GFP at the indicated MOI for 2 h, and unbound virus was washed away later.

The enterovirus 71 Xiangyang strain (GenBank accession no. JN230523.1) was isolated previously by our group (47). Cells were infected with EV71 at the indicated MOI after serum starvation overnight, and unbound virus was washed away 2 h later (48).

### Luciferase assay.

The determination of reporter luciferase activity was performed as described previously (49). In brief, cells were transfected with the indicated plasmids and a luciferase reporter plasmid for 24 h in 293T cells or for 48 h in HepG2 cells and then washed twice with ice-cold PBS. One hundred microliters of luciferase lysis buffer (Promega, Madison, WI, USA) was added to each well of a 24-well plate. Cells were lysed for 10 min at room temperature, and 50 μl of each sample was then transferred to a new centrifuge tube and mixed with 15 μl luciferase assay substrate (Promega, Madison, WI, USA). Luciferase activity was typically measured for 10 s after a 3-s delay by using a luminometer (TD-20/20; Turner Designs, Sunnyvale, CA, USA). All assays were performed in triplicate, and data are expressed as means ± standard deviations (SD).

### Antibodies and reagents.

Antibodies against Flag (catalog no. F1804) and hemagglutinin (HA) (catalog no. H6908) were purchased from Sigma-Aldrich (St. Louis, MO, USA). Antibodies against IFNAR1 (catalog no. ab45172) and NSE1 (catalog no. ab139689) were purchased from Abcam (Cambridge, MA, USA). Antibody against IRAK1 (catalog no. 4504S) was purchased from Cell Signaling Technology (CST) (Beverly, MA, USA). Antibodies against GAPDH (catalog no. 60004-1-Ig), GFP (catalog no. 66002-1-Ig), GST (catalog no. 66001-1-Ig), SMC5 (catalog no. 14178-1-AP), SMC6 (catalog no. 14465-1-AP), and PJA1 (catalog no. 17687-1-AP) were purchased from Proteintech Group (Wuhan, Hubei, China). Antibody against NSMCE4A (catalog no. AP9909a) was purchased from Abgent (San Diego, CA, USA).

Hepatitis B virus antigen detection ELISA kits were purchased from Ke Hua Bio Engineering (Shanghai, China). The inhibitors Braco19 and MG132 were purchased from Sigma-Aldrich. 3-Deazaneplanocin A (DZNeP), 5-aza-2ʹ-deoxycytidine (5′-Aza), GSK343, idarubicin HCl, and topotecan HCl were purchased from Selleck Chemicals (Houston, TX, USA).

### Plasmids and constructions.

The luciferase reporters were kindly provided by Ying Zhu of Wuhan University. The firefly luciferase coding sequence (CDS) was constructed in pLenti-CMV-GFP by replacing the sequence encoding EGFP to generate pLenti-CMV-Luciferase for lentivirus production. PJA1 cDNA was generously provided by Jiahuai Han of Xiamen University and constructed in pCAGGS-HA, containing a C-terminal HA tag, to generate pHA-PJA1. PJA1B and the RING domain deletion mutants PJA1ΔR and PJA1BΔR were subcloned into the same vector. The full-length and truncated PJA1 genes were constructed in pcDNA3.1 to generate tag-free proteins used for competitive co-IP and function verification. The PJA1B CDS was constructed in pLenti-CMV-3Flag to generate pLenti-CMV-PJA1B for lentivirus production. SMC5/6 and NSE1/3/4 coding sequences were amplified by PCR from 293T cell cDNA and constructed in pcDNA3.1-3Flag, containing an N-terminal 3×Flag tag. The NSE4 CDS was constructed in pCAGGS-HA to generate pHA-NSE4.

HBV-1.3(*ayw*) was generated from HepG2.2.15 cells (genotype D, subtype *ayw*; GenBank accession no. U95551) and inserted into pBluescript II (Invitrogen). The short hairpin RNAs (shRNAs) used for gene knockdown were cloned into pLKO.1. The target sequences were as follows: 5′-GTGAGCAGCTTTGTAAACGAAT-3′ for sh-SMC6 and 5′-GCGAGGAGTGACCAAGTGAAA-3′ for sh-PJA1. Small interfering RNA (siRNA) oligonucleotides specific for human topoisomerases were synthesized by Guangzhou RiboBio (Guangzhou, China) using the primers 5′-AAGGACTCCATCAGATACTAT-3′ for siR-TOP1, 5′-GCCCAAGTGTTCTTTAGCTTT-3′ for siR-TOP2a, and 5′-AGAAGAGTCAGAAACTGCC-3′ for siR-TOP2b.

All plasmids were confirmed by sequencing analyses, and the resulting proteins were verified by Western blotting or luciferase activity.

### Cell proliferation assay.

Cell proliferation was examined with a cell counting kit 8 (CCK-8) assay according to the manufacturer's instructions (MedChem Express, USA). In brief, cells were plated in a 96-well plate at a density of 1,000 cells/well in 100 μl of culture medium. Ten microliters of the CCK-8 solution was added to each well of the plate, and the plate was incubated for 2 h at 37°C in an incubator. Viable cell numbers were estimated by measurement of the absorbance at 450 nm. All assays were performed in triplicate, and data are expressed as means ± SD.

### Coimmunoprecipitation.

In assays with transfected cells, 2 × 10^6^ cells were lysed in 500 μl radioimmunoprecipitation assay (RIPA) lysis buffer (250 mM Tris-HCl, 150 mM NaCl, 1 mM EDTA, 1% NP-40, 5% glycerol [pH 7.4]). Five percent of the lysate was removed for input. Cell lysates were precleared by using protein G-agarose beads (GE Healthcare) for 1 h at 4°C, mixed with specific antibodies or the corresponding IgG and protein G-agarose beads, and rotated overnight at 4°C. Beads were washed 5 times by resuspending them in 1 ml 150 mM NaCl wash buffer and incubating them for 5 min on a rotating platform at 4°C between each wash, resuspended and denatured in PBS with SDS-PAGE loading buffer, and then separated by SDS-PAGE, with subsequent immunoblot analysis.

### GST pulldown.

GST, GST-NSE3, GST-NSE4, and MBP-PJA1BΔR were expressed by the E. coli BL21 strain and purified. The cell extracts were prepared by ultrasonication and incubated with glutathione-Sepharose beads (GE Healthcare). The GST-, GST-NSE3-, and GST-NSE4-immobilized beads were washed 5 times with ice-cold PBS. The resulting immobilized beads were incubated with MBP-PJA1BΔR lysates at 4°C for 1 h, washed 5 times with cold PBS, denatured with SDS-PAGE loading buffer, and analyzed by SDS-PAGE and immunoblotting.

### Chromatin immunoprecipitation assay.

Cells were harvested and fixed with 1% formaldehyde for 10 min. The formaldehyde was quenched by the addition of cold glycine at a final concentration of 125 mM for 5 min and then washed twice with ice-cold PBS. Cells were resuspended in sucrose lysis buffer (10 mM Tris-HCl [pH 8.0], 50 mM NaCl, 10 mM EDTA, 20% sucrose, 1× protease and phosphatase inhibitor cocktail) for 15 min. After the addition of an equal amount of 2× IP buffer (200 mM Tris-HCl [pH 8.0], 600 mM NaCl, 4% Triton X-100, 2× protease and phosphatase inhibitor cocktail), lysates were sonicated on ice, and debris were removed by centrifugation at 10,000 × *g* for 15 min at 4°C. Five percent of the supernatant was used as the DNA input. The remaining supernatant was diluted 10-fold with dilution buffer (1% Triton X-100, 2 mM EDTA, 20 mM Tris-HCl [pH 8.0], and 150 mM NaCl), incubated with primary antibody, and rotated overnight at 4°C. Immunoprecipitated complexes were collected by using protein G-agarose beads. The pellets were washed once with low-salt wash buffer (0.1% SDS, 1% Triton X-100, 2 mM EDTA, 20 mM Tris-HCl, 150 mM NaCl), once with high-salt wash buffer (0.1% SDS, 1% Triton X-100, 2 mM EDTA, 20 mM Tris-HCl, 500 mM NaCl), and twice with Tris-EDTA buffer and incubated at 65°C for 4 h to reverse the formaldehyde cross-link. After RNase A and proteinase K digestion, precipitated DNA was purified using the E.Z.N.A. Cycle-Pure kit (catalog no. D6492; Omega).

### Real-time qPCR.

Total RNA was extracted from cells with TRIzol reagent (Invitrogen Life Technologies) according to the manufacturer's instructions. DNA was removed from the sample using RNase-free DNase I (Promega) treatment at 37°C for 30 min in the presence of 1 U/μl RNasin (Promega). Real-time quantitative PCR (RT-qPCR) was performed by using SYBR green PCR master mix in a LightCycler 480 thermal cycler (Roche). Endogenous GAPDH was used as an internal control. The relative amount of amplified product was calculated using the comparative threshold cycle (*C_T_*) method.

The following primers were used: 5′-AAGGCTGTGGGCAAGG-3′ (forward) and 5′-TGGAGGAGTGGGTGTCG-3′ (reverse) for GAPDH, 5′-GATGGGTTAGGAGTGGCTGAA-3′ (forward) and 5′-ACGGCTTGTGGAAATAGTGG-3′ (reverse) for PJA1, 5′-CTGAACCTTTACCCCGTTGC-3′ (forward) and 5′-GCGGGATAGGACAACAGAGT-3′ (reverse) for HBV DNA, 5′-GAGTGTGGATTCGCACTCC-3′ (forward) and 5′-GAGGCGAGGGAGTTCTTCT-3′ (reverse) for HBV pgRNA, 5′-GCATCCTTCGTGTTTGTCATT-3′ (forward) and 5′-GCATCTTCTCTCCGACCCCG-3′ (reverse) for ICP27, 5′-CTTCAGATGGCTTCGAGATCGTAG-3′ (forward) and 5′-TGTTTACTTAAAAGGCGTGCCGT-3′ (reverse) for US11, 5′-TTTCTCCTGCCTGAAGGACAG-3′ (forward) and 5′-GCTCATGATTTCTGCTCTGACA-3′ (reverse) for IFN-α, 5′-GCCGCATTGACCATGTATGAGA-3′ (forward) and 5′-GAGATCTTCAGTTTCGGAGGTAAC-3′ (reverse) for IFN-β, 5′-GTGGAGACCATCAAGGAAGACA-3′ (forward) and 5′-TATTGCAGGCAGGACAACCA-3′ (reverse) for IFN-γ, 5′-AAAGCGAACAAGGAGTAAG-3′ (forward) and 5′-GATGATGCCATCCCGTAG-3′ (reverse) for double-stranded RNA-activated protein (PKR), 5′-TTCCGTCCATAGGAGCCAC-3′ (forward) and 5′-AAGCCCTACGAAGAATGTC-3′ (reverse) for OAS1, and 5′-TTCAGCACCTGATGGCCTATC-3′ (forward) and 5′-TGGATGATCAAAGGGATGTGG-3′ (reverse) for MX1.

The following primers were used for chromatin immunoprecipitation (ChIP) followed by real-time qPCR: 5′-AAGGGCGTCGAAACTGTCAT-3′ (forward) and 5′-GCAAGTTGTCCGAAACCGAC-3′ (reverse) for UL36, 5′-GCCGCCCCGTACCTCGTGAC-3′ (forward) and 5′-CAGCCCGCTCCGCTTCTCG-3′ (reverse) for pVP16, and 5′-TCGCCAGTCAAGTAACAAC-3′ (forward) and 5′-ACTTCGTCCACAAACACAA-3′ (reverse) for luciferase.

### Immunofluorescence and confocal analyses.

HepG2 cells were grown in glass-bottom dishes. Twenty-four hours after transfection, cells were fixed with 4% paraformaldehyde for 15 min, washed three times with PBS, permeabilized with PBS containing 0.2% Triton X-100 for 5 min, washed three times with PBS, and blocked with PBS containing 10% bovine serum albumin for 30 min at room temperature. The cells were then incubated with anti-HA and anti-Flag overnight at 4°C, followed by incubation with cy3-conjugated goat anti-rabbit IgG and fluorescein isothiocyanate (FITC)-conjugated goat anti-mouse IgG for 45 min at room temperature, and stained with DAPI (4′,6-diamidino-2-phenylindole). Cells were viewed by confocal laser microscopy (FluoView FV1000; Olympus).

### Statistical analysis.

The results are presented as means ± SD. Student's *t* test for paired samples was used to determine statistical significance. Statistical testing was performed using Prism 5 software (GraphPad Software Inc.). Differences were considered statistically significant at a *P* value of ≤0.05.

## References

[1] El-Serag HB 2012 Epidemiology of viral hepatitis and hepatocellular carcinoma. Gastroenterology 142:1264–1273. doi:10.1053/j.gastro.2011.12.061. 22537432 PMC3338949

[2] Ganem D, Varmus HE 1987 The molecular biology of the hepatitis B viruses. Annu Rev Biochem 56:651–693. doi:10.1146/annurev.bi.56.070187.003251. 3039907

[3] Roizman B, Knipe DM, Whitley RJ 2013 Herpes simplex viruses, p 1823–1897. *In* Knipe DM, Howley PM, Cohen JI, Griffin DE, Lamb RA, Martin MA, Racaniello VR, Roizman B (ed), Fields virology, 6th ed Lippincott Williams & Wilkins, Philadelphia, PA.

[4] Schacker T 2001 The role of HSV in the transmission and progression of HIV. Herpes 8:46–59. 11867018

[5] Yoon WJ, Cho YD, Cho KH, Woo KM, Baek JH, Cho JY, Kim GS, Ryoo HM 2008 The Boston-type craniosynostosis mutation MSX2 (P148H) results in enhanced susceptibility of MSX2 to ubiquitin-dependent degradation. J Biol Chem 283:32751–32761. doi:10.1074/jbc.M803183200. 18786927

[6] Yu P, Chen Y, Tagle DA, Cai T 2002 PJA1, encoding a RING-H2 finger ubiquitin ligase, is a novel human X chromosome gene abundantly expressed in brain. Genomics 79:869–874. doi:10.1006/geno.2002.6770. 12036302

[7] Stork O, Stork S, Pape HC, Obata K 2001 Identification of genes expressed in the amygdala during the formation of fear memory. Learn Mem 8:2091–2099. doi:10.1101/lm.39401. PMC311378 11533224

[8] Jakubiczka S, Collmann H, Zuffardi O, Zackai E, Wieacker P 2007 Contiguous gene deletions involving EFNB1, OPHN1, PJA1 and EDA in patients with craniofrontonasal syndrome. Clin Genet 72:506–156. doi:10.1111/j.1399-0004.2007.00905.x. 17941886

[9] Consalvi S, Brancaccio A, Dall'Agnese A, Puri PL, Palacios D 2017 Praja1 E3 ubiquitin ligase promotes skeletal myogenesis through degradation of EZH2 upon p38α activation. Nat Commun 8:13956. doi:10.1038/ncomms13956. 28067271 PMC5423270

[10] Shin J, Mishra V, Glasgow E, Zaidi S, Ohshiro K, Chitti B, Kapadia AA, Rana N, Mishra L, Deng CX, Rao S, Mishra B 2017 PRAJA is overexpressed in glioblastoma and contributes to neural precursor development. Genes Cancer 8:640–649. doi:10.18632/genesandcancer.151. 28966725 PMC5620009

[11] Loch CM, Eddins MJ, Strickler JE 2011 Protein microarrays for the identification of praja1 e3 ubiquitin ligase substrates. Cell Biochem Biophys 60:127–135. doi:10.1007/s12013-011-9180-x. 21461837

[12] Sasaki A, Masuda Y, Iwai K, Ikeda K, Watanabe K 2002 A RING finger protein Praja1 regulates Dlx5-dependent transcription through its ubiquitin ligase activity for the Dlx/Msx-interacting MAGE/Necdin family protein, Dlxin-1. J Biol Chem 277:22541–22546. doi:10.1074/jbc.M109728200. 11959851

[13] Zoabi M, Sadeh R, de Bie P, Marquez VE, Ciechanover A 2011 PRAJA1 is a ubiquitin ligase for the polycomb repressive complex 2 proteins. Biochem Biophys Res Commun 408:393–398. doi:10.1016/j.bbrc.2011.04.025. 21513699

[14] Taylor EM, Moghraby JS, Lees JH, Smit B, Moens PB, Lehmann AR 2001 Characterization of a novel human SMC heterodimer homologous to the Schizosaccharomyces pombe Rad18/Spr18 complex. Mol Biol Cell 12:1583–1594. doi:10.1091/mbc.12.6.1583. 11408570 PMC37326

[15] Uhlmann F 2016 SMC complexes: from DNA to chromosomes. Nat Rev Mol Cell Biol 17:399–412. doi:10.1038/nrm.2016.30. 27075410

[16] Hu B, Liao C, Millson SH, Mollapour M, Prodromou C, Pearl LH, Piper PW, Panaretou B 2005 Qri2/Nse4, a component of the essential Smc5/6 DNA repair complex. Mol Microbiol 55:1735–1750. doi:10.1111/j.1365-2958.2005.04531.x. 15752197

[17] Pebernard S, McDonald WH, Pavlova Y, Yates JR III, Boddy MN 2004 Nse1, Nse2, and a novel subunit of the Smc5-Smc6 complex, Nse3, play a crucial role in meiosis. Mol Biol Cell 15:4866–4876. doi:10.1091/mbc.e04-05-0436. 15331764 PMC524734

[18] Pebernard S, Wohlschlegel J, McDonald WH, Yates JR III, Boddy MN 2006 The Nse5-Nse6 dimer mediates DNA repair roles of the Smc5-Smc6 complex. Mol Cell Biol 26:1617–1630. doi:10.1128/MCB.26.5.1617-1630.2006. 16478984 PMC1430260

[19] Alt A, Dang HQ, Wells OS, Polo LM, Smith MA, McGregor GA, Welte T, Lehmann AR, Pearl LH, Murray JM, Oliver AW 2017 Specialized interfaces of Smc5/6 control hinge stability and DNA association. Nat Commun 8:14011. doi:10.1038/ncomms14011. 28134253 PMC5290277

[20] Hwang G, Sun F, O'Brien M, Eppig JJ, Handel MA, Jordan PW 2017 SMC5/6 is required for the formation of segregation-competent bivalent chromosomes during meiosis I in mouse oocytes. Development 144:1648–1660. doi:10.1242/dev.145607. 28302748 PMC5450844

[21] Fujioka Y, Kimata Y, Nomaguchi K, Watanabe K, Kohno K 2002 Identification of a novel non-structural maintenance of chromosomes (SMC) component of the SMC5-SMC6 complex involved in DNA repair. J Biol Chem 277:21585–21591. doi:10.1074/jbc.M201523200. 11927594

[22] Torres-Rosell J, Machín F, Farmer S, Jarmuz A, Eydmann T, Dalgaard JZ, Aragón L 2005 SMC5 and SMC6 genes are required for the segregation of repetitive chromosome regions. Nat Cell Biol 7:412–419. doi:10.1038/ncb1239. 15793567

[23] Decorsiere A, Mueller H, van Breugel PC, Abdul F, Gerossier L, Beran RK, Livingston CM, Niu C, Fletcher SP, Hantz O, Strubin M 2016 Hepatitis B virus X protein identifies the Smc5/6 complex as a host restriction factor. Nature 531:386–380. doi:10.1038/nature17170. 26983541

[24] Mitra B, Guo H 2016 Hepatitis B virus X protein crosses out Smc5/6 complex to maintain covalently closed circular DNA transcription. Hepatology 64:2246–2249. doi:10.1002/hep.28834. 27639252 PMC5115954

[25] Murphy CM, Xu Y, Li F, Nio K, Reszka-Blanco N, Li X, Wu Y, Yu Y, Xiong Y, Su L 2016 Hepatitis B virus X protein promotes degradation of SMC5/6 to enhance HBV replication. Cell Rep 16:2846–2854. doi:10.1016/j.celrep.2016.08.026. 27626656 PMC5078993

[26] Champoux JJ 2001 DNA topoisomerases: structure, function, and mechanism. Annu Rev Biochem 70:369–413. doi:10.1146/annurev.biochem.70.1.369. 11395412

[27] Uemura T, Yanagida M 1984 Isolation of type I and II DNA topoisomerase mutants from fission yeast: single and double mutants show different phenotypes in cell growth and chromatin organization. EMBO J 3:1737–1744. doi:10.1002/j.1460-2075.1984.tb02040.x. 6090122 PMC557590

[28] Wang JC 2002 Cellular roles of DNA topoisomerases: a molecular perspective. Nat Rev Mol Cell Biol 3:430–440. doi:10.1038/nrm831. 12042765

[29] Kanno T, Berta DG, Sjogren C 2015 The Smc5/6 complex is an ATP-dependent intermolecular DNA linker. Cell Rep 12:1471–1482. doi:10.1016/j.celrep.2015.07.048. 26299966

[30] Rai R, Laloraya S 2017 Genetic evidence for functional interaction of Smc5/6 complex and Top1 with spatial frequency of replication origins required for maintenance of chromosome stability. Curr Genet 63:765–776. doi:10.1007/s00294-017-0680-0. 28204881

[31] Mlcochova P, Caswell SJ, Taylor IA, Towers GJ, Gupta RK 2018 DNA damage induced by topoisomerase inhibitors activates SAMHD1 and blocks HIV-1 infection of macrophages. EMBO J 37:50–62. doi:10.15252/embj.201796880. 29084722 PMC5753034

[32] Paludan SR, Bowie AG 2013 Immune sensing of DNA. Immunity 38:870–880. doi:10.1016/j.immuni.2013.05.004. 23706668 PMC3683625

[33] Huttlin EL, Bruckner RJ, Paulo JA, Cannon JR, Ting L, Baltier K, Colby G, Gebreab F, Gygi MP, Parzen H, Szpyt J, Tam S, Zarraga G, Pontano-Vaites L, Swarup S, White AE, Schweppe DK, Rad R, Erickson BK, Obar RA, Guruharsha KG, Li K, Artavanis-Tsakonas S, Gygi SP, Harper JW 2017 Architecture of the human interactome defines protein communities and disease networks. Nature 545:505–509. doi:10.1038/nature22366. 28514442 PMC5531611

[34] Zabrady K, Adamus M, Vondrova L, Liao C, Skoupilova H, Novakova M, Jurcisinova L, Alt A, Oliver AW, Lehmann AR, Palecek JJ 2016 Chromatin association of the SMC5/6 complex is dependent on binding of its NSE3 subunit to DNA. Nucleic Acids Res 44:1064–1079. doi:10.1093/nar/gkv1021. 26446992 PMC4756808

[35] Pebernard S, Perry JJ, Tainer JA, Boddy MN 2008 Nse1 RING-like domain supports functions of the Smc5-Smc6 holocomplex in genome stability. Mol Biol Cell 19:4099–4109. doi:10.1091/mbc.e08-02-0226. 18667531 PMC2555936

[36] Taylor EM, Copsey AC, Hudson JJ, Vidot S, Lehmann AR 2008 Identification of the proteins, including MAGEG1, that make up the human SMC5-6 protein complex. Mol Cell Biol 28:1197–1206. doi:10.1128/MCB.00767-07. 18086888 PMC2258758

[37] Barbalat R, Ewald SE, Mouchess ML, Barton GM 2011 Nucleic acid recognition by the innate immune system. Annu Rev Immunol 29:185–214. doi:10.1146/annurev-immunol-031210-101340. 21219183

[38] Wu J, Chen ZJ 2014 Innate immune sensing and signaling of cytosolic nucleic acids. Annu Rev Immunol 32:461–488. doi:10.1146/annurev-immunol-032713-120156. 24655297

[39] Niu C, Livingston CM, Li L, Beran RK, Daffis S, Ramakrishnan D, Burdette D, Peiser L, Salas E, Ramos H, Yu M, Cheng G, Strubin M, Delaney WE IV, Fletcher SP 2017 The Smc5/6 complex restricts HBV when localized to ND10 without inducing an innate immune response and is counteracted by the HBV X protein shortly after infection. PLoS One 12:e0169648. doi:10.1371/journal.pone.0169648. 28095508 PMC5240991

[40] Doyle JM, Gao J, Wang J, Yang M, Potts PR 2010 MAGE-RING protein complexes comprise a family of E3 ubiquitin ligases. Mol Cell 39:963–974. doi:10.1016/j.molcel.2010.08.029. 20864041 PMC4509788

[41] Feng Y, Gao J, Yang M 2011 When MAGE meets RING: insights into biological functions of MAGE proteins. Protein Cell 2:7–12. doi:10.1007/s13238-011-1002-9. 21337005 PMC4875283

[42] Lee AK, Potts PR 2017 A comprehensive guide to the MAGE family of ubiquitin ligases. J Mol Biol 429:1114–1142. doi:10.1016/j.jmb.2017.03.005. 28300603 PMC5421567

[43] Bermudez-Lopez M, Ceschia A, de Piccoli G, Colomina N, Pasero P, Aragón L, Torres-Rosell J 2010 The Smc5/6 complex is required for dissolution of DNA-mediated sister chromatid linkages. Nucleic Acids Res 38:6502–6512. doi:10.1093/nar/gkq546. 20571088 PMC2965248

[44] Tapia-Alveal C, Outwin EA, Trempolec N, Dziadkowiec D, Murray JM, O'Connell MJ 2010 SMC complexes and topoisomerase II work together so that sister chromatids can work apart. Cell Cycle 9:2065–2070. doi:10.4161/cc.9.11.11734. 20495382

[45] Ni Y, Lempp FA, Mehrle S, Nkongolo S, Kaufman C, Fälth M, Stindt J, Königer C, Nassal M, Kubitz R, Sültmann H, Urban S 2014 Hepatitis B and D viruses exploit sodium taurocholate co-transporting polypeptide for species-specific entry into hepatocytes. Gastroenterology 146:1070–1083. doi:10.1053/j.gastro.2013.12.024. 24361467

[46] Knipe DM, Spang AE 1982 Definition of a series of stages in the association of two herpesviral proteins with the cell nucleus. J Virol 43:314–324. 6287005 10.1128/jvi.43.1.314-324.1982 PMC256122

[47] Song Y, Cheng X, Yang X, Zhao R, Wang P, Han Y, Luo Z, Cao Y, Zhu C, Xiong Y, Liu Y, Wu K, Wu J 2015 Early growth response-1 facilitates enterovirus 71 replication by direct binding to the viral genome RNA. Int J Biochem Cell Biol 62:36–46. doi:10.1016/j.biocel.2015.02.012. 25724735

[48] Luo Z, Ge M, Chen J, Geng Q, Tian M, Qiao Z, Bai L, Zhang Q, Zhu C, Xiong Y, Wu K, Liu F, Liu Y, Wu J 2017 HRS plays an important role for TLR7 signaling to orchestrate inflammation and innate immunity upon EV71 infection. PLoS Pathog 13:e1006585. doi:10.1371/journal.ppat.1006585. 28854257 PMC5595348

[49] Chen Y, Chen J, Wang H, Shi J, Wu K, Liu S, Liu Y, Wu J 2013 HCV-induced miR-21 contributes to evasion of host immune system by targeting MyD88 and IRAK1. PLoS Pathog 9:e1003248. doi:10.1371/journal.ppat.1003248. 23633945 PMC3635988

[50] Teuber J, Mueller B, Fukabori R, Lang D, Albrecht A, Stork O 2013 The ubiquitin ligase Praja1 reduces NRAGE expression and inhibits neuronal differentiation of PC12 cells. PLoS One 8(5):e63067. doi:10.1371/journal.pone.0063067. 23717400 PMC3661586

